# Historical Biogeography of the Marine Snail *Littorina saxatilis* Inferred from Haplotype and Shell Morphology Evolution in NW Spain

**DOI:** 10.1371/journal.pone.0161287

**Published:** 2016-08-11

**Authors:** Terencia Tirado, María Saura, Emilio Rolán-Alvarez, Humberto Quesada

**Affiliations:** 1 Departamento de Bioquímica, Genética e Inmunología, Facultad de Biología, Universidad de Vigo, Vigo, Spain; 2 Departamento de Mejora Genética Animal, INIA, Madrid, Spain; Australian Museum, AUSTRALIA

## Abstract

The marine snail *Littorina saxatilis* exhibits extreme morphological variation between and within geographical regions and represents an excellent model for assessing local adaptation. Previous studies support the hypothesis of parallel evolution in sympatry of two morphologically different ecotypes (named as RB and SU) that co-inhabit different habitats from Galician rocky shores (NW Spain), and which are interrupted by sheltered areas inhabited by a different morph never studied before (named as SRB). Here, we use morphological and mitochondrial DNA (mtDNA) sequence data to test hypotheses on the origin and diversification of SRB snails and to assess their evolutionary relationships with RB and SU ecotypes. Our results show that the SRB morph displays the largest size and shell elongation and the smallest relative shell aperture, representing an extreme type of the RB vs. SU polymorphism, which has been linked to adaptation to sheltered ecological factors. Phylogenetic analysis shows that the SRB morph shares ancestry with RB and SU ecotypes, rejecting the hypothesis that the SRB morph marks relict populations from which these ecotypes evolved in Galician coasts. Our data support that genetic differentiation among SRB, RB and SU morphs results from a general pattern of restricted gene flow and isolation by distance linked to the colonization of Galician coasts by two independent mtDNA lineages, rather than from a random fragmentation of the initial distributional range. Therefore, the confinement of distinct lineages to specific geographical areas denote evident limits to the distances these snails can disperse. Morphological analysis indicates no association between mtDNA lineage and a specific morphotype, and suggests the independent gain of convergent morphological patterns within each mtDNA lineage in populations occupying contrasting habitats following the colonization of Galician coasts.

## Introduction

Ecological speciation is the process by which barriers to gene flow evolve between populations as a result of ecologically-based divergent selection [1–3]. The marine gastropod *Littorina saxatilis* is an example of incomplete ecological speciation that can be observed along the wave exposed-rocky shores in Galicia (NW Spain) (reviewed in [4]). Galician populations of this organism display an extreme microhabitat-associated intraspecific dimorphism in response to different environmental conditions. The SU (smooth and unbanded shell) ecotype lives in the lower shore on mussels (exposed microhabitat), where the main factor affecting survival is the strength of the waves. The large and robust RB (ridged and banded) ecotype lives in the upper shore associated with barnacles (sheltered microhabitat), where it is exposed to daily changes in salinity, heat stress and higher predation rates by crabs. These ecotypes show partial reproductive isolation and a low dispersal capacity [4]. In the midshore, both habitats overlap and in some areas, the two ecotypes meet and occasionally mate producing fertile intermediate morphological forms (hybrids) [5–6]. A similar pattern of pairs of divergent ecotypes currently living in contrasting habitats can be found in the west coast of Sweden and the northern coast of England [4, 7].

Two hypotheses have been proposed to explain the origin of the above ecotypes: I) divergence in allopatry followed by secondary contact, and II) parallel non-allopatric evolution of the ecotypes at both regional and local scales [4, 8, 9]. Results from a recent study by Butlin et al. [10] combining data from three geographical regions (Spain, England and Sweden) using mitochondrial and nuclear data gave additional support to the hypothesis that the ecotypes arose in parallel and in sympatry [9]. This paper showed, using an Approximate Bayesian Computation framework, that pairs of ecotypes likely arose independently in the face of continuous gene flow both within and between European regions, after a long delay between the colonization of a locality and ecotype formation. The authors also found that Galician populations have been historically isolated from Northern Europe, as the estimated time of separation between the northern and Spanish populations was much older than between British and Swedish populations (see [11–12]).

Although RB and SU ecotypes have been extensively studied (reviewed in [4]), questions underlying this incipient speciation process still remain unanswered. The ecotypes and their habitats are found exclusively in the most exposed areas of the Galician coast. These exposed sites are interrupted by sheltered areas. Populations inhabiting these sheltered areas have never been studied before and they may contain residual populations with a phylogenetic signal that might shed light on the importance of allopatric and non-allopatric processes on the origin of RB and SU ecotypes (see [13–14]).

Numerous studies have successfully used mitochondrial DNA (mtDNA) for assessing phylogenetic relationships in presence of gene flow to elucidate the relative importance of historical and ecological factors on intraspecific geographic variation (e.g. [15–18]). Therefore, mtDNA evolves rapidly and is unlikely to recombine, thus becoming a highly informative marker for the formulation of unambiguous phylogenetic hypotheses and the resolution of recent radiations among taxa [15]. In *L*. *saxatilis*, previous work [4, 9, 10] has validated the analysis of mtDNA to perform evolutionary inferences in Galician populations of this marine snail, as it mirrors evolutionary inferences based on multilocus nuclear markers assayed at the same sites. Further studies have also stressed its utility to disentangle the evolutionary history of this species in other geographical regions [11–12]. A global molecular phylogeny revealed the existence of two independent mtDNA lineages (I and II) resulting from an ancient divergence event around the British Isles that pre-dated the formation of European ecotypes 2.5–13 kya by at least 0.6 Myr [10–11]. These lineages have experienced subsequent diversification into a number of derived haplogroups and are now broadly distributed across the North Atlantic region, including Galician coasts [11].

Here, we analyze mtDNA sequences as well as phenotypic data (shell morphometrics) of samples from both exposed shores (inhabited by RB and SU ecotypes) and sheltered shores (inhabited by the putative sheltered ecotype, SRB, henceforth) from Galicia covering the Spanish distribution range of the species. We aimed to distinguish between three main scenarios. In scenario A, the SRB morph is the relict form from which RB and SU ecotypes evolved in Galician coasts. Under this scenario, haplotypes from all the sheltered sites should cluster together in phylogenetic trees into a separated and highly diverged clade owing to their ancestral condition. In scenario B, genetic differentiation among SRB, RB and SU morphs results from a general pattern of restricted gene flow and isolation by distance linked to the colonization of Galician coasts by at least two independent mtDNA lineages (I and II). Under this hypothesis, haplotypes should cluster in phylogenetic trees by regional geographical origin, with a positive relationship between geographical and genetic distance within each major mtDNA lineage. In scenario C, an initial population bearing at least two divergent mtDNA lineages (I and II) and lacking population structure experienced vicariance events that had a location and timing independent of the geographical distribution of *L*. *saxatilis* snails. Under this hypothesis, haplotypes from each morph and lineage would cluster essentially randomly within the phylogeny.

## Materials and Methods

### Sampling

A total of 48 adult snails inhabiting the most sheltered areas (the sheltered SRB morph) from four Galician estuaries were sampled covering the distribution range of the species in Spain (Table 1). We also used data from the exposed ecotypes (SU and RB) from a previous study by Quesada et al. [9], which included four exposed Galician localities where RB and SU individuals coexist in sympatry (Table 1). This study did not involve endangered or protected species, and no specific permission for collection of specimens was required.

**Table 1:** Sampling sites of Galician (NW Spain) *L*. *saxatilis*.

Region	Locality	Latitude / Longitude	Ecotype	N
	Mogor	42° 23´ 22.5” N / 8° 42´56.9”W	SRB	12
Inner Bay	Noia	42° 47´30.2” N / 8° 54´36.2” W	SRB	12
	Sada	43° 21´20.3” N / 8° 14´59.8” W	SRB	12
	Tapia	43° 34´20.2" N / 6° 56´50.0” W	SRB	12
	Cíes	42° 13´29.8” N / 8° 54´20.0” W	RB	6
Outer Bay^1^	Ons	42° 22´31.6” N / 8° 55´55.8” W	RB	6
	Roncudo	43° 17´32.7” N / 8° 57´30.9” W	RB	6
	Arealonga	43° 45´10.6” N / 7° 38´12.0” W	RB	6
	Cíes	42° 13´29.8” N / 8° 54´20.0” W	SU	6
Outer Bay^1^	Ons	42° 22´31.6” N / 8° 55´55.8” W	SU	6
	Roncudo	43° 17´32.7” N / 8° 57´30.9” W	SU	6
	Arealonga	43° 45´10.6” N / 7° 38´12.0” W	SU	6

^1^From Quesada et al. [9]

### Morphometric analysis

Each snail was photographed with a Leica MZ12 stereoscopic microscope and Leica digital ICA video camera. Adult shell images were analyzed using 11 landmarks positioned on the digitized shell image following Conde-Padín et al. [19]. For each individual, we measured centroid size (CS) and shape using relative warps (RW). The relative warps were computed using the software packages TpsDig and TpsRelw [20–21], excluding the uniform component, following Butlin et al. [10]. We used the scaling option α = 0, which weights all landmarks equally. Differences among ecotypes were interpreted for the consensus form using a thin-plate spline representation, an interpolating function to describe shape changes with respect to the reference configuration [20], using the TpsRelw software.

We first applied a randomization ANOVA in order to detect differences attributable to ecotypes ignoring their locality of origin, as suggested by Peres-Neto and Olden [22]. Second, we evaluated the occurrence of differences in shell size and shape among the three ecotypes (RB, SU and SRB) by a two-way nested parametric ANOVA, with factors *morph* (fixed: SRB, RB and SU) and *locality* (nested within morph, random: three levels for each morph), following Underwood [23]. Analyses were performed using the statistical package SPSS 12.0.

### DNA extraction and sequencing

Head–foot tissue was used for DNA extraction, using a CTAB protocol [24]. DNA concentration and purity were assessed using a NanoDrop spectrophotometer (Nanodrop Tech. Inc., Wilmington, DE). DNA samples were purified with NucleoSpin columns following the manufacturer’s instructions (Macherey-Nagel). All DNA samples were standardized to 50 ng/μL.

Primers used in this study are those developed by Butlin et al. [10], designed from the annotated *L*. *saxatilis* partial mtDNA sequence (AJ132137; [25]) and from sequences in Small and Gosling [26]. These primers were used to amplify a 2004 bp region (in two overlapping fragments of 1028 and 1137 bp) encompassing the *ND6* and t*RNApro* mitochondrial genes, as well as the 3´end of the *ND1* gene and the 5´end of the *Cyt-b* gene [10]. After PCR purification using GFX columns (Amersham Biosciences, Picataway, NJ), sequencing was performed for both strands in an ABI 3730 xl sequencer. Newly reported mtDNA haplotypes were deposited in the EMBL database under accession numbers LT593763-LT593842.

### Sequence analysis

Contigs were assembled using Seqman (DNASTAR, Madison, WI) and alignments were performed with ClustalX [27]. Alignments were manually inspected for ambiguities in Bioedit 7.2.2 [28]. Data were searched for evidence of recombination using the program RDP4 [29]. Polymorphism estimates were calculated with DnaSp 5.10.1 [30]. The raggedness index *r* [31] and the Fu´s *Fs* statistic [32] were computed to test for population expansions. Statistical significance of these tests was evaluated by coalescent simulations with no recombination as implemented in DnaSp. We performed an analysis of molecular variance (AMOVA) considering a two-level hierarchical partition (localities and ecotypes) using Arlequin 3.5.1.2. [33]. This program was also used for calculating population pairwise F_ST_ estimates. The program zt [34] was used to compute a Mantel test between the linearized F_ST_ estimates [F_ST_ /(1- F_ST_)] and log-transformed geographical distances between samples.

The optimal nucleotide substitution model was selected based on the Akaike Information Criterion in Modeltest 3.7 [35]. The selected model (HKY85) was then used to perform a maximum-likelihood phylogeny as implemented in PhyML [36] and including sequences from the Spanish RB and SU ecotypes from the study by Quesada et al. [9]. Node confidence was assessed by running 1000 bootstrap replicates. We also carried out a Bayesian phylogenetic analysis using MrBAYES 3.1 [37]. Four chains were run for 8 x 10^6^ generations using default values and trees saved every 1000 generations. The initial 2 x 10^6^ generations were discarded for burn-in. The analysis was repeated twice and checked for convergence. In addition, a statistical parsimony network [38] was built up with Network 4.6 [39] using the median-joining algorithm.

The program PhyloMapper v.1b1 [40] was used to test the null hypothesis that the inferred clade structure reflects a random geographic association of lineages. This program uses a maximum likelihood approach for parameter estimation given the geographical coordinates of the individuals represented by tips on the tree. We randomized the assignment of geographical locations to the tips of the genealogy 10,000 times. If several localities shared a haplotype, then we randomly selected one of these localities for analysis. The distribution of the dispersal parameter (*Ψ*) obtained from this randomization process was used as an indicator of phylogeographic association [40], and compared to the value of *Ψ* obtained when the relationship between the genealogy and sampling localities was not randomized.

We used a constrained tree analysis as implemented in RAxML [41] to better distinguish alternative evolutionary scenarios. A maximum likelihood search was performed in RAxML for each of three tree topologies: (1) unconstrained tree; (2) constrained tree with monophyly of sheltered SRB populations; and (3) constrained tree with monophyly of populations living in the same locality. The likelihoods of constrained trees were compared to the reference unconstrained tree using the Shimodaira-Hasegawa test [42] as implemented in Tree-Puzzle 5.3 [43].

## Results

**Fig 1:**
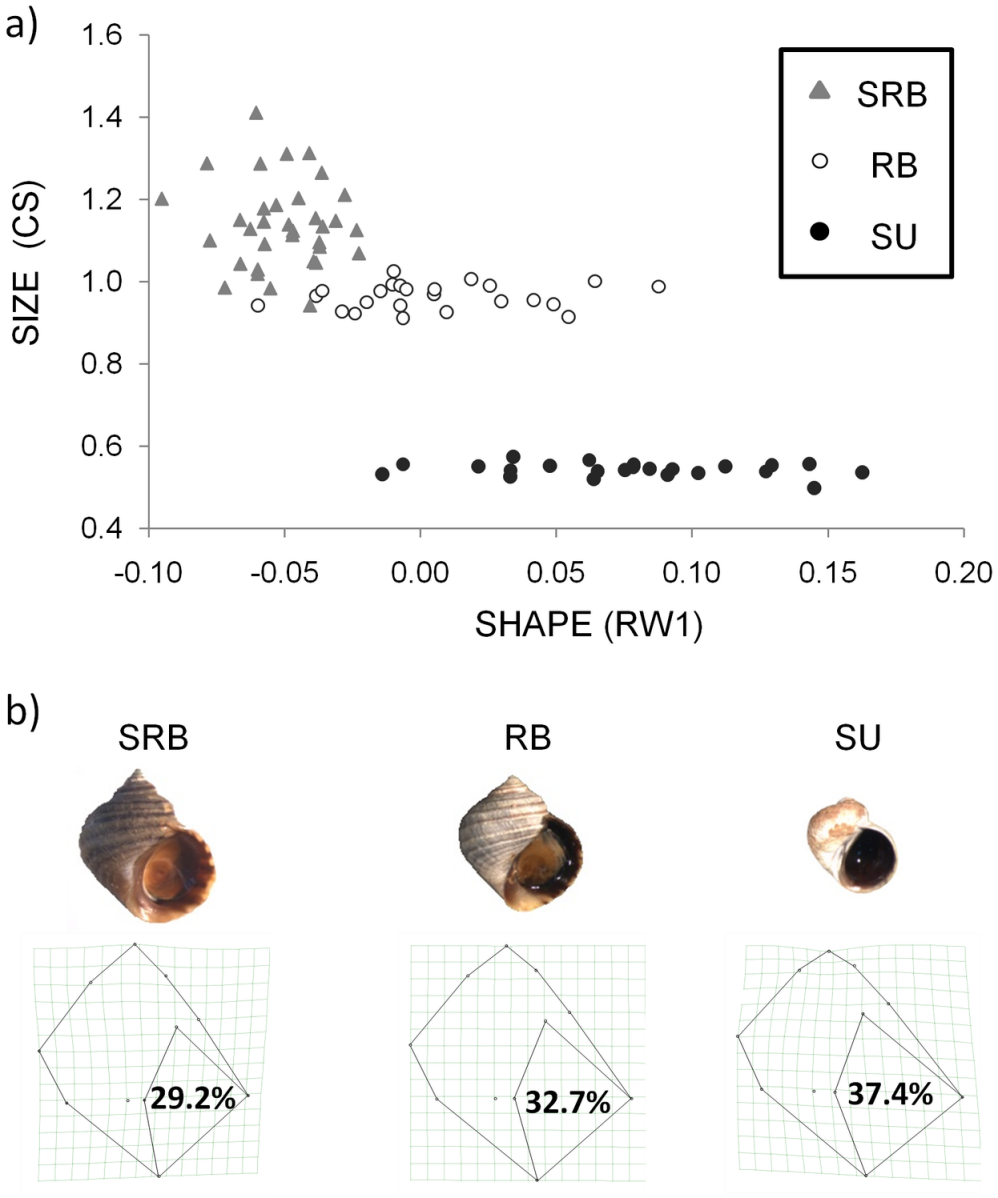
Geometric morphometrics analysis. a) Individuals from different ecotypes (SRB, RB and SU) and localities plotted for size (CS) and the relative shell aperture (RW1) of shell shape. b) Photo and corresponding thin-plate spline representation of the geometric deformations explained by RW1 for the three ecotypes (SRB, RB and SU). Percentages correspond to aperture size values.

Morphology was consistent across regions within each of the three morphs studied but differed among morphs. Both the randomization and the nested ANOVA revealed the occurrence of significant differences in size (CS) and shape (U1, RW1) among morphs. According to the results derived from the nested ANOVA, the 91% (CS), 39% (U1) and 67% (RW1) of the morphological variation observed for each variable was due to differences among morphs. The means (and standard deviations) of these variables for each of the morphs and localities in addition to the results of the ANOVAS are summarized in Table 2. A clear differentiation between the three morphs was observed for CS, reflecting that SRB (1.152±0.120) displays a significantly larger size than RB (0.964±0.031) and SU (0.544±0.016) morphs. The nested factor *locality* was also significant for CS and RW1, but the percentage of variance explained was lower than 16% (data not shown), thus suggesting that although the differences between morphs were not identical across localities, these differences might be minor. Morphological differences between pairs of morphs were always statistically significant using a post-hoc Student–Newman–Keuls (SNK) tests for U1, RW1 and CS. The magnitude, however, was not always identical. For example, the difference in relative aperture size (RW1) was somewhat larger between RB and SU than between RB and SRB (see Fig 1). In summary, the morphotypes showed a clear shell differentiation in relation to size (CS), longitudinal elongation (U1) and relative shell aperture (RW1). The SRB morph had the largest size, the smallest relative shell aperture, and most elongated shells, in contrast to the opposite extreme represented by the SU ecotype (Fig 1).

**Table 2:** Mean (±SD) of the significant variables derived from the geometric morphometrics analyses. Results are given for each morphotype and locality, including the results from both the randomization (ignoring the origin of the localities) and the nested (including the geographical origin) ANOVAS, indicating the existence of significant differences among morphotypes.

					Morphometric variables^1^		
Region	Ecotype	Habitat	Locality	N	CS	U1	RW1
	SRB	Supersheltered	Mogor	12	1.138 ± 0.071	0.021 ± 0.020	0.051 ± 0.028
Inner Bay	SRB	Supersheltered	Noia	12	1.249 ± 0.132	0.024 ± 0.025	−0.062 ± 0.025
	SRB	Supersheltered	Sada	12	1.068 ± 0.071	0.020 ± 0.015	0.050 ± 0.011
	SRB	Supersheltered	Tapia	12	NA	NA	NA
	RB	Sheltered	Cíes	6	0.960 ± 0.031	0.001 ± 0.015	0.054 ± 0.020
Outer Bay	RB	Sheltered	Ons	6	0.992 ± 0.018	0.002 ± 0.023	0.110 ± 0.014
	RB	Sheltered	Roncudo	6	0.945 ± 0.030	0.030 ± 0.015	0.009 ± 0.023
	RB	Sheltered	Arealonga	6	0.961 ± 0.029	0.005 ± 0.030	0.015± 0.028
	SU	Exposed	Cíes	6	0.534 ± 0.021	0.007 ± 0.028	0.095 ± 0.033
Outer Bay	SU	Exposed	Ons	6	0.544 ± 0.011	0.035 ± 0.032	0.107 ± 0.028
	SU	Exposed	Roncudo	6	0.551 ± 0.013	0.036 ± 0.039	0.079 ± 0.050
	SU	Exposed	Arealonga	6	0.546 ± 0.015	0.024 ± 0.031	0.026 ± 0.030
Randomization ANOVA					410.7***	26.9***	104.3***
Nested ANOVA					92.0***	17.1**	12.0**

^1^ CS: size; U1: elongation (parallel to the axis of coiling); RW1: relative shell aperture.

***p≤ 0.001;

**p≤ 0.01.

NA: not available

DNA sequence data revealed a substantial level of variation in the assayed mtDNA fragment among the 47 sheltered SRB specimens examined. There were 20 different haplotypes in the sheltered SRB populations, with a total of 59 variable sites. No evidence was found of genetic recombination for the entire data set (N = 95) including RB, SU and the 47 newly generated SRB mtDNA sequences. We observed no significant differences in overall nucleotide diversity between SRB (π = 0.0067 ± 0.0006), RB (π = 0.0078 ± 0.0009) and SU (π = 0.0079 ± 0.0010) morphotypes (see S1 Table). Levels of genetic diversity at single localities did not show any latitudinal pattern of variation (*r*^2^ = 0.104; *P* = 0.435). No evidence of population expansion was found at any site, as the raggedness *r* index and Fu´s *Fs* were never statistically significant.

Genetic variation was highly geographically structured among localities. All pairwise comparisons among morphotypes from different localities resulted in a highly significant (*P* < 0.001) genetic differentiation, with F_ST_ values ranging from 0.971 (Cíes SU *vs* Arealonga RB) to 0.191 (Cíes SU *vs* Ons SU). Geographical distance was positively correlated with the level of genetic differentiation (*r* = 0.295; *P* = 0.001, Mantel test) but not with morphological divergence (*r* = -0.161, *P* = 0.171). A hierarchical analysis of molecular variance revealed no significant (*P* = 0.858) differences in the level of geographic differentiation of SRB, SU and RB ecotypes, whereas most of molecular variance (89%) was explained by differences between localities within each ecotype (*P* < 0.001).

**Fig 2:**
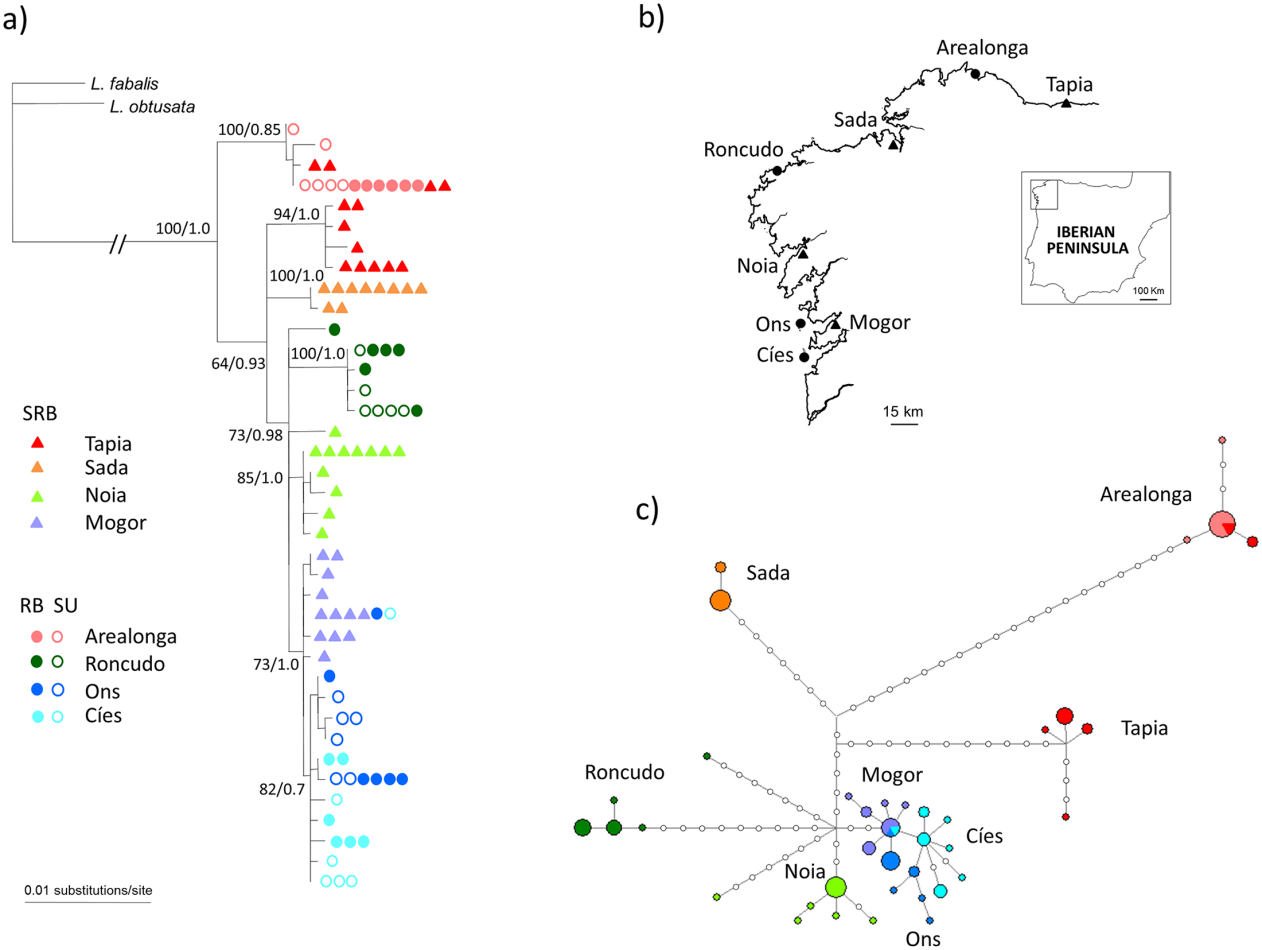
Phylogenetic relationships among Galician snails. (a) Phylogram of Galician *L*. *saxatilis*. Numbers above branches indicate bootstrap node support in RAxML/Bayesian posterior probability in MrBAYES. Only bootstrap values and posterior probabilities of, respectively, 60% and 0.7 or greater are depicted. (b) Location of sampling sites. (c) Statistical parsimony network. Pie charts represent the frequencies at each locality. Each white circle represents a mutational step. Note that clade I specimens from Tapia are clustered together with Arealonga clade I specimens in the same pie chart. In all panels, colour and symbol designation indicates geographic origin.

The phylogenetic analysis clearly indicated a global pattern in which haplotypes clustered according to their regional geographical origin with a high node support (Fig 2a). Bayesian and maximum likelihood methods provided congruent tree topologies, with no disagreement between nodes supported with bootstrap values and posterior probabilities greater than or equal to 60% and 0.7 respectively. Results from PhyloMapper strongly rejected a random geographic distribution of phylogenetic clades (*Ψ* = 28.11; *P* < 0.001). The geographically proximate Ons, Cíes and Mogor populations shared two different haplotypes and formed a separate and well defined monophyletic clade. Interestingly, two haplotypes found at low-frequency in Tapia most likely represent a recent event of gene flow from the nearby location Arealonga. The Shimodaira-Hasegawa (SH) test assuming monophyly of individuals from the same location (ln L = -858.63) was not significantly different from the unconstrained tree (Ln L = -854.17; SH-test, *P* = 0.505). In contrast, a constrained tree topology assuming that all SRB populations cluster in a single monophyletic clade was significantly different from the unconstrained tree (Ln L = -927.05, SH test, *P* < 0.001).

The haplotype network (Fig 2c) contained a strong signal of local radiations in which haplotypes arise from a small number of divergent haplotypes in the same population. To explore the relationships between Galician and North Atlantic *L*. *saxatilis* snails, we added data from a previous study by Doellman et al. [11], which included 32 populations (from western, central and eastern North Atlantic) using a much smaller mtDNA fragment of 1154 bp (accession numbers AM500966-AM500967). The haplotype network (S1 Fig) inferred from this shorter mtDNA region provided a lower resolution for Galician populations and revealed, as previously reported, that *L*. *saxatilis* is composed of two major and broadly distributed mtDNA lineages (I and II) [11]. Galician localities displayed independent ranges of lineages I and II. Thus, except individuals originating from Arealonga and a few from Tapia that formed a monophyletic subgroup within lineage I, individuals from all other Galician localities were included within lineage II, also forming a separate monophyletic subgroup. No haplotypes were shared with non-Galician populations, as expected from a long-term isolation of Galician snails from their northern counterparts. Clade structure for Galician snails within lineage II confirmed a non-random geographic association (*Ψ* = 23.15; *P* < 0.001) and a significant correlation between geographical distance and genetic differentiation (*r* = 0.433; *P* < 0.001, Mantel test). This analysis was not feasible for lineage I due to its small clade size.

The results above allow us to reject the scenario A, which predicts a major and ancestral monophyletic clade including all sheltered SRB individuals and scenario C, which predicts that haplotypes from each morph and lineage would cluster essentially randomly within the phylogeny. Instead, our results agree with scenario B, because this hypothesis predicts the monophyly of haplotypes from the same regional geographical origin, and a general pattern of restricted gene flow and isolation by distance linked to the colonization of Galician coasts by two independent mtDNA lineages.

## Discussion

In this study, we presented morphological and mitochondrial DNA (mtDNA) sequence data to test hypotheses on the origin and diversification of the intertidal snail *Littorina saxatilis* inhabiting the Galician coast (NW Spain). Our results indicate a clear morphological distinctness of the SRB morph (Fig 1 and Table 2). Phylogenetic analysis (Fig 2) shows that the SRB morph shares ancestry with RB and SU ecotypes, rejecting the hypothesis that the SRB morph marks relict populations from which these ecotypes evolved in Galician coasts. Our data support that genetic differentiation among SRB, RB and SU morphs results from a general pattern of restricted gene flow and isolation by distance linked to the colonization of Galician coasts by two independent mtDNA lineages, rather than from a random fragmentation of the initial distributional range. Therefore, the confinement of distinct lineages to specific geographical areas indicate evident limits to the distances these snails can disperse. These results are of particular relevance for SRB snails living inside the Galician estuaries, which have not been examined before the current study, and that could have harboured refugia populations.

Our data confirm the previous claim that Galician *L*. *saxatilis* mtDNA is in fact polyphyletic, including derived haplotypes from two ancient lineages (I and II) originated around the British Isles about 0.64 Ma [11]. The split into two major lineages is well supported since haplotypes of populations newly investigated compared to the previous mtDNA study by Doellman et al. [11] belonged to only two haplogroups, clustering either in the I or in the II lineage without forming a new one (S1 Fig). Our study provides a more exhaustive sampling scheme allowing to accurately pinpoint the geographical range of each lineage in Galician coasts. Lineage I is geographically restricted to a narrow northern region, whereas lineage II represents the common source of haplotypes for the majority of populations from Galician coasts (S1 Fig). This finding explains the previously reported high divergence between the northern location of Arealonga and other southern Galician populations [9], since they belong to different haplogroups (S1 Fig).

The observation of two major ancient mtDNA lineages complicates the former identification of scenarios of repeated evolution [9], because the emergence of distinct ecotype pairs in sympatry on exposed Galician shores may be confounded by admixture of divergent genetic lineages. However, given the mtDNA topology and the lack of an association of lineages I and II with a specific morphological phenotype, there is no simple way to explain the morphological divergence between morphotypes as old and predating colonization, unless we also invoke 1) a long-term stabilizing selection to preserve ancestral morphological characters in widely separated and genetically divergent morphotypes, 2) repeated episodes of extinction of the native mtDNA lineage of one ecotype and its subsequent replacement by the alternative lineage, and 3) that these mtDNA replacements have occurred in opposite directions in Northern and Southern Galician populations. A more plausible explanation seems to be the independent gain of convergent morphological patterns in independent genetic lineages in response to similar environmental gradients [9, 10].

Our data give small support to dramatic influences of Quaternary climate changes on Galician populations. The absence of latitudinal patterns of genetic diversity, the relatively high haplotype diversity, the nonexistence of genetic differences in average diversity among morphotypes, and the strong population structure including isolation by distance, support that Galician populations have remained relatively stable and isolated after colonization of the different regions and localities. A relatively long-term stable demographic scenario is in agreement with the fact that the Iberian Peninsula was ice-free during the last glacial maximum [44], and that Galician *L*. *saxatilis* populations have been historically isolated from those living in northern areas, heavily affected by population size fluctuations and dramatic changes in distributional ranges [10–12]. This strengthens the view that Galician populations display unique genetic patterns that could have facilitated a steady long-term accumulation of local adaptive differences [4, 9, 10]. Accordingly, earlier studies based on allozyme, AFLP and genome-wide DNA sequencing analyses have emphasized the role that local selection may play in structuring genetic variation within and among natural populations of Galician *L*. *saxatilis* (reviewed in [4]). For example Galindo et al. [45] reported that up to 3% of AFLP loci examined in ecotype pairs of this species living in the same Galician exposed site are directly or indirectly affected by directional selection in response to the specific habitats where they live. Similarly, RNA-seq and RAD data have provided widespread evidence for adaptive changes specific to the particular habitat where *L*. *saxatilis* ecotypes inhabit [46–47].

Our results demonstrate for the first time the morphological distinctness of SRB snails. This is particularly interesting because the shell size and shape differences existing between the RB and SU morphs have been previously interpreted in adaptive terms (reviewed in [4] for CS and RW1), for which a genetic basis with a small contribution from developmental plasticity has been demonstrated [5, 19, 48, 49]. Additionally, two different snail species (*Nucella lapillus* and *Melarraphe neritoides*) showed a similar parallel trend in shell shape (RW1) across the same environmental cline [50]. The SRB morph lives in the most sheltered habitats and shows the most extreme sheltered morphological traits of all Galician morphotypes reported so far, displaying the largest shells and smallest apertures (Fig 1). This could either suggest an adaptation to withstand the crab predation, desiccation, and the heat stress, or any plastic response caused by growing in the upper shore level.

In conclusion, the results of our study suggest that the colonization of Galician coasts by ancestral *L*. *saxatilis* populations bearing two divergent mtDNA lineages was an important process helping to explain the current variation in the distribution of mtDNA haplogroups. Our morphological and phylogenetic analyses indicate no association between mtDNA lineage and a specific morphotype, and do not support the hypothesis that the SRB morph marks relict populations. The distribution of mtDNA clades may, therefore, represent an additional example of the importance of restricted gene flow and isolation by distance in explaining patterns of genetic divergence in this species. More generally, we provide an example of parallel phenotypes that have evolved across multiple genetic lineages. Altogether, the conclusions derived from this study will provide baseline data for future research on the amazing capacity of this species to reach morphological changes in response to any kind of intertidal microhabitat [4, 7].

## Supporting Information

S1 Fig Relationships between Galician and North Atlantic *L*. *saxatilis* snails. (a) Statistical parsimony network for North Atlantic populations of *L*. *saxatilis*. Data include Galician and 32 additional populations (from western, central and eastern North Atlantic) using a much smaller mtDNA fragment. Grey indicates *L*. *arcana*; black indicates *L*. *compressa*. Haplogroup designations (A to J) and colours as in Doellman et al. [11]. Galician haplotypes are enclosed within a green line (haplogroup D from lineage I) and a red line (haplogroup H from lineage II). (b) Haplotype frequencies for Galician *L*. *saxatilis*. In Galicia, only two haplogroups were found: haplogroup D from lineage I (in green), and haplogroup H from lineage II (in red).
(PDF)

S1 Table Summary of the mtDNA diversity in Galician *L*. *saxatilis*. Estimates of mtDNA nucleotide variation for each ecotype and locality of Galician *L*. *saxatilis*
(PDF)

## References

[1] Rundle HD, Nosil P. Ecological speciation. Ecology Letters. 2005; 8: 336–352.

[2] Schluter D. Evidence for ecological speciation and its alternative. Science. 2009; 323: 737–741. 10.1126/science.1160006 19197053

[3] Nosil P. Ecological speciation Oxford University Press, Oxford, UK; 2012.

[4] Rolán-Alvarez E, Austin C, Boulding EG. The contribution of *Littorina* to the field of Evolutionary Ecology. Oceanography and Marine Biology: an Annual Review. 2015; 53: 157–214.

[5] Galindo J, Martínez-Fernández M, Rodríguez-Ramilo S, Rolán-Alvarez E. The role of local ecology during hybridisation at the initial stages of ecological speciation in a marine snail. J Evol Biol. 2013; 26: 1472–1487. 10.1111/jeb.12152 23663115

[6] Galindo J, Rivas MJ, Saura M, Rolán-Alvarez E. Selection on hybrids of ecologically divergent ecotypes of a marine snail: the relative importance of exogenous and endogenous barriers. Biol J Linn Soc. 2014; 111: 391–400.

[7] Johannsesson K. What can be learnt from a snail? Evol Appl. 2015 10.1111/eva.12277 PMC4780386 27087845

[8] Rolán-Alvarez E, Carballo M, Galindo J, Morán P, Fernández B, Caballero A, et al Non-allopatric and parallel origin of local reproductive barriers between two snail ecotypes. Mol Ecol. 2004; 13: 3415–3424. 15488000 10.1111/j.1365-294X.2004.02330.x

[9] Quesada H, Posada D, Caballero A, Morán P, Rolán-Alvarez E. Phylogenetic evidence for multiple sympatric ecological diversification in a marine snail. Evolution. 2007; 61: 1600–1612. 17598743 10.1111/j.1558-5646.2007.00135.x

[10] Butlin R, Saura M, Charrier G, Jackson B, Andre C, Caballero A, et al Parallel local adaptation in the face of gene flow. Evolution. 2014; 68: 935–949. 24299519 10.1111/evo.12329 PMC4261988

[11] Doellman MM, Trussell GC, Grahame JW, Vollmer SV. Phylogeographic analysis reveals a deep lineage split within North Atlantic *Littorina saxatilis*. Proc R Soc London B. 2011; 278: 3175–3183. 10.1098/rspb.2011.0346 PMC3169032 21429920

[12] Panova M, Blakeslee AMH, Miller AW, Mäkinen T, Ruiz GM, Johannesson K, et al Glacial history of the North Atlantic marine snail, *Littorina saxatilis*, inferred from distribution of mitochondrial DNA lineages. PLoS ONE. 2011; 6: e17511 10.1371/journal.pone.0017511 21412417 PMC3055875

[13] Bierne N, Welch J, Loire E, Bonhomme F, David P. The coupling hypothesis: why genome scans may fail to map local adaptation genes. Mol Ecol. 2011; 20: 2044–2072. 10.1111/j.1365-294X.2011.05080.x 21476991

[14] Bierne N, Gagnaire PA, David P. The geography of introgression in a patchy environment and the thorn in the side of ecological speciation. Curr Zool. 2013; 59: 72–86.

[15] Brower AVZ. Rapid morphological radiation and convergence among races of the butterfly *Heliconious erato* inferred from patterns of mitochondrial DNA evolution. Proc Natl Acad Sci USA. 1994; 91: 6491–6495. 8022810 10.1073/pnas.91.14.6491 PMC44228

[16] Civois A, Thibault JC, Pasquet E. Uniform phenotype conceals double colonization by reed-warblers of a remote Pacific archipielago. J Biogeogr. 2007; 34: 1150–1166.

[17] Guiller A, Madec L. Historical biogeography of the land snail *Cornu aspersum*: a new scenario inferred from haplotype distribution in the Western Mediterranean basin. BMC Evol Biol. 2010; 10: 18 10.1186/1471-2148-10-18 20089175 PMC2826328

[18] Dennemoser S, Nolte AW, Vamosi SM, Rogers SM. Phylogeography of the prickly sculpin (*Cottus asper*) in north-western North America reveals parallel phenotypic evolution across multiple coastal-inland colonizations. J Biogeogr. 2015; 42: 1626–1638.

[19] Conde-Padín P, Caballero A, Rolán-Alvarez E. Relative role of genetic determination and plastic response during ontogeny for shell-shape traits subjected to diversifying selection. Evolution. 2009; 63: 1356–1363. 10.1111/j.1558-5646.2009.00636.x 19187255

[20] Rohlf FJ. TPSRELW: relative warp analysis. Department of Ecology and Evolution, State University of New York, Stony Brook, NY; 2005.

[21] Rohlf FJ. TPSDIG: a program for digitizing “landmarks” and outliers for geometric morphometric analyses. Department of Ecology and Evolution, State University of New York, Stony Brook, NY; 2006.

[22] Peres-Neto PR, Olden JD. Assessing the robustness of randomization test: examples from behavioural studies. Anim Behav. 2001; 61: 79–86. 11170698 10.1006/anbe.2000.1576

[23] Underwood AJ. Techniques of analysis of variance in experimental marine biology and ecology. Ocean Mar Biol Ann Rev. 1981; 19: 513–605.

[24] Wilding CS, Butlin RK, Grahame JW. Differential gene exchange between parapatric morphs of *Littorina saxatilis* detected using AFLP markers. J Evol Biol. 2001; 14: 611–619.

[25] Wilding CS, Mill PJ, Grahame JW. Partial sequence of the mitochondrial genome of *Littorina saxatilis*: relevance to gastropod phylogenetics. Journal of Molecular Evolution. 1999; 48: 348–359. 10093225 10.1007/pl00006479

[26] Small MP, Gosling EM. Genetic structure and relationships in the snail species complex *Littorina arcana* Hannaford and Ellis, *L*. *compressa* Jeffreys and *L*. *saxatilis* (Olivi) in the British Isles using SSCPs of cytochrome-b fragments. Heredity. 2000; 84: 692–701. 10886385 10.1046/j.1365-2540.2000.00717.x

[27] Thompson JD. The CLUSTAL_X window interface: flexible strategies for multiple sequence alignment aided by quality analysis tools. Nucl Acids Res. 1997; 25: 4876–4882. 9396791 10.1093/nar/25.24.4876 PMC147148

[28] Hall TA. BioEdit: a user-friendly biological sequence alignment editor and analysis program for Windows 95/98/NT. Nucleic Acids Symp Ser. 1999; 41: 95–98.

[29] Martin DP, Murrell B, Golden M, Khoosal A, Muhire B. RDP4: Detection and analysis of recombination patterns in virus genomes. Virus Evol; 2015; 1 10.1093/ve/vev003 PMC5014473 27774277

[30] Librado P, Rozas J. DnaSP v5: A software for comprehensive analysis of DNA polymorphism data. Bioinformatics. 2009; 25: 1451–1452. 10.1093/bioinformatics/btp187 19346325

[31] Harpending HC. Signature of ancient population growth in a low-resolution mitochondrial-DNA mismatch distribution. Human Biology. 1994; 66: 591–600. 8088750

[32] Fu Y-X. Statistical tests of neutrality of mutations against population growth, hitchhiking and background selection. Genetics. 1997; 147: 915–925. 9335623 10.1093/genetics/147.2.915 PMC1208208

[33] Excoffier L, Lischer HEL. Arlequin suite ver 3.5: A new series of programs to perform population genetics analyses under Linux and Windows. Mol Ecol Res. 2010; 10: 564–567. 10.1111/j.1755-0998.2010.02847.x 21565059

[34] Bonnet E, Van de Peer Y. zt: a software tool for simple and partial Mantel tests. J Stat Softw. 2002; 7: 1–12.

[35] Posada D, Crandal KA. Modeltest: testing the model of DNA substitution. Bioinformatics. 1998; 14: 817–818. 9918953 10.1093/bioinformatics/14.9.817

[36] Guindon S, Gascuel O. A simple, fast, and accurate algorithm to estimate large phylogenies by maximum likelihood. Syst Biol. 2003; 52: 696–704. 14530136 10.1080/10635150390235520

[37] Huelsenbeck JP, Ronquist F. MRBAYES: Bayesian inference of phylogenetic trees. Bioinformatics. 2001; 17: 754–755. 11524383 10.1093/bioinformatics/17.8.754

[38] Templeton AR, Crandall KA, Sing CF. A cladistic analysis of phenotypic associations with haplotypes inferred from restriction endonuclease mapping and DNA sequence data. III. Cladogram estimation. Genetics. 1992; 132: 619–633. 1385266 10.1093/genetics/132.2.619 PMC1205162

[39] Bandelt H-J, Forster P, Röhl A. Median-joining networks for inferring intraspecific phylogenies. Mol Biol Evol. 1999; 16: 37–48. 10331250 10.1093/oxfordjournals.molbev.a026036

[40] Lemmon AR, Lemmon EM. A likelihood framework for estimating phylogeographic history on a continuous landscape. Sys Biol. 2008; 57: 544–561. 10.1080/10635150802304761 18686193

[41] Stamakakis A, Hoover P, Rougemont J. A rapid bootstrap algorithm for the RAxML Web-Servers. Syst Biol. 2008; 75: 758–771. 10.1080/10635150802429642 18853362

[42] Shimodaira H, Hasegawa M. Multiple comparisons of log-likelihoods with applications to phylogenetic inference. Mol Biol Evol. 1999; 16: 1114–1116.

[43] Schmidt HA, Strimmer K, Vingron M, von Haeseler A. TREE-PUZZLE: maximum likelihood phylogenetic analysis using quartets and parallel computing. Bioinformatics. 2002; 18: 502–504 11934758 10.1093/bioinformatics/18.3.502

[44] Taberlet P, Fumagalli L, Wust-Saucy A-G, Cosson J-F. Comparative phylogeography and postglacial colonization routes in Europe. Mol Ecol. 1998; 7: 453–464. 9628000 10.1046/j.1365-294x.1998.00289.x

[45] Galindo J, Morán P, Rolán-Alvarez E. Comparing geographical genetic differentiation between candidate and noncandidate loci for adaptation strengthens support for parallel ecological divergence in the marine snail *Littorina saxatilis*. Mol Ecol. 2009; 18: 919–930 10.1111/j.1365-294X.2008.04076.x 19207246

[46] Westram AM, Galindo J, Alm Rosenblad M, Grahame JW, Panova M. Do the same genes underlie parallel phenotypic divergence in different *Littorina saxatilis* populations? Mol Ecol. 2014; 23: 4603–4616. 10.1111/mec.12883 25113130 PMC4285301

[47] Ravinet M, Westram A, Johannesson K, Butlin R, André C, Panova M. Shared and non-shared genomic divergence in parallel ecotypes of *Littorina saxatilis* at a local scale. Mol Ecol. 2015; 10.1111/mec.13332 26222268

[48] Johannesson K, Johannsesson B, Rolán-Alvarez E. Morphological differentiation and genetic cohesiveness over a microenvironmental gradient in the marine snail *Littorina saxatilis*. Evolution. 1993; 47: 1770–1787. 28568008 10.1111/j.1558-5646.1993.tb01268.x

[49] Johannsesson K, Rolán-Alvarez E, Erlandsson K. Growth rate differences between upper and lower shore ecotypes of the marine snail *Littorina saxatilis* (Olivi) (Gastropoda). Biol J L Soc. 1997; 61: 267–279.

[50] Cuña V, Saura M, Quesada H, Rolán-Álvarez E. Extensive micro-geographical shell polymorphism in a planktotrophic marine intertidal snail. Mar Ecol Prog Ser. 2011; 427: 133–143.

