# Supplementary material for: Historical Biogeography of the Marine Snail Littorina saxatilis Inferred from Haplotype and Shell Morphology Evolution in NW Spain
Source: PLoS One. 2016 Aug 11;11(8):e0161287. doi: 10.1371/journal.pone.0161287 (PMC4981350; doi:10.1371/journal.pone.0161287)
Supplement: S1 Fig — (a) Statistical parsimony network for North Atlantic populations of L. saxatilis. Data include Galician and 32 additional populations (from western, central and eastern North Atlantic) using a much smaller mtDNA fragment. Grey indicates L. arcana; black indicates L. compressa. Haplogroup designations (A to J) and colours as in Doellman et al. [11]. Galician haplotypes are enclosed within a green line (haplogroup D from lineage I) and a red line (haplogroup H from lineage II). (b) Haplotype frequencies for Galician L. saxatilis. In Galicia, only two haplogroups were found: haplogroup D from lineage I (in green), and haplogroup H from lineage II (in red). (PDF) [file pone.0161287.s001.pdf]

SUPPORTING INFORMATION

Historical biogeography of the marine snail *Littorina saxatilis* inferred from haplotype and shell morphology evolution in NW Spain

Terencia Tirado, María Saura, Emilio Rolán-Alvarez and Humberto Quesada

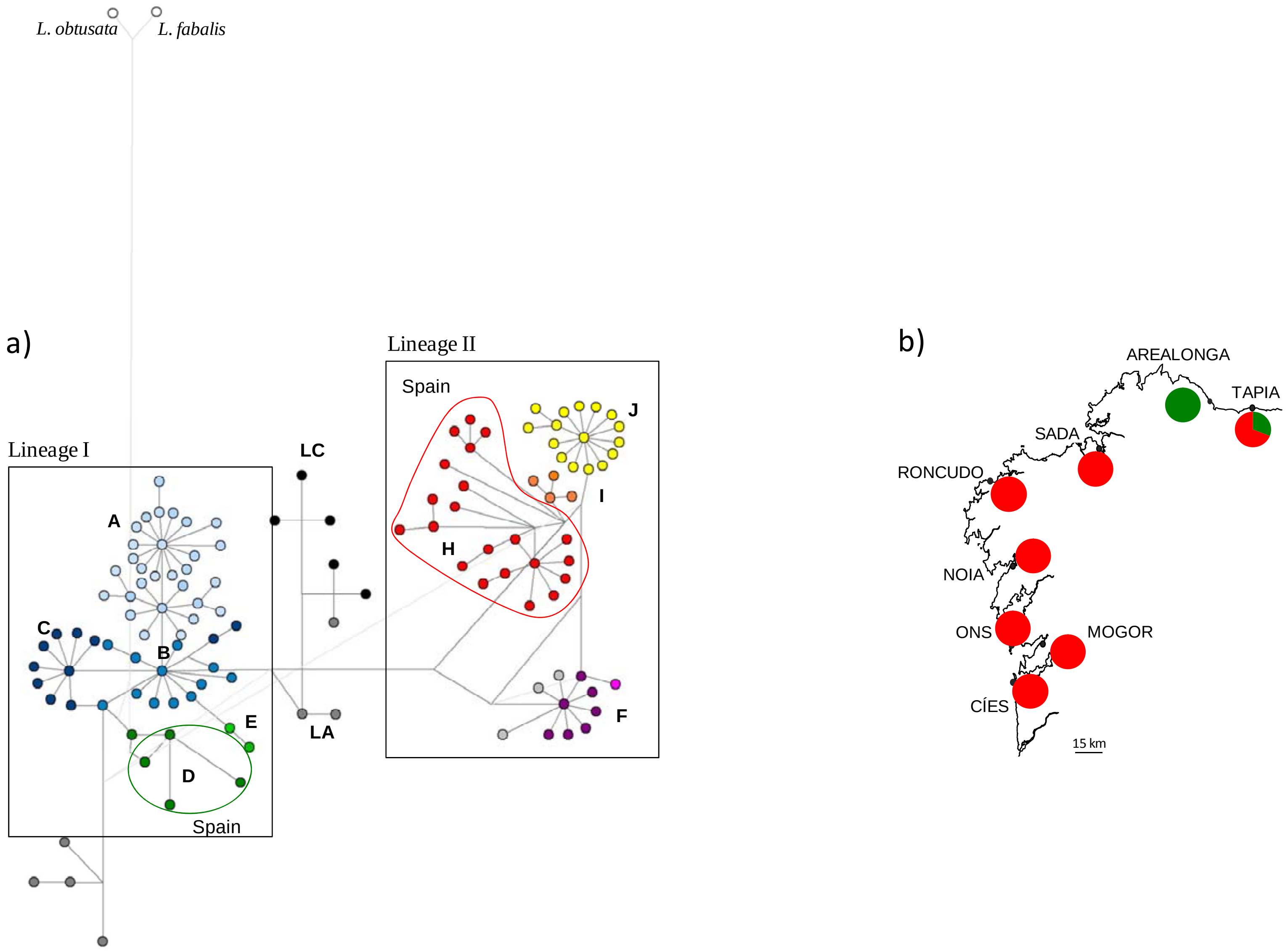

S1 Fig. (a) Statistical parsimony network for North Atlantic populations of *L. saxatilis.* Data include Galician and 32 additional populations (from western, central and eastern North Atlantic) using a much smaller mtDNA fragment. Grey indicates *L. arcana*; black indicates *L. compressa*. Haplogroup designations (A to J) and colours as in Doellman et al. [11]. Galician haplotypes are enclosed within a green line (haplogroup D from lineage I) and a red line (haplogroup H from lineage II). (b) Haplotype frequencies for Galician *L. saxatilis*. In Galicia, only two haplogroups were found: haplogroup D from lineage I (in green), and haplogroup H from lineage II (in red).
