# Supplementary material for: Historical Biogeography of the Marine Snail Littorina saxatilis Inferred from Haplotype and Shell Morphology Evolution in NW Spain
Source: PLoS One. 2016 Aug 11;11(8):e0161287. doi: 10.1371/journal.pone.0161287 (PMC4981350; doi:10.1371/journal.pone.0161287)
Supplement: S1 Table — Estimates of mtDNA nucleotide variation for each ecotype and locality of Galician L. saxatilis (PDF) [file pone.0161287.s002.pdf]

SUPPORTING INFORMATION

Historical biogeography of the marine snail *Littorina saxatilis* inferred from haplotype and shell morphology evolution in NW Spain

Terencia Tirado, María Saura, Emilio Rolán-Alvarez and Humberto Quesada

S1 Table. Estimates of mtDNA nucleotide variation for each ecotype and locality of Galician *L. saxatilis*

Region	Ecotype	Habitat	Locality	N	S[a]	Hd[b]	π[c]
	SRB	Supersheltered	Mogor	12	5	0.848 ± 0.071	0.0007 ± 0.0001
Inner Bay	SRB	Supersheltered	Noia	12	12	0.682 ± 0.148	0.0013 ± 0.0005
	SRB	Supersheltered	Sada	10	1	0.356 ± 0.159	0.0002 ± 0.0001
	SRB	Supersheltered	Tapia	13	30	0.833 ± 0.081	0.0070 ± 0.0014
			**TOTAL**	**47**	**59**	**0.932 ± 0.018**	**0.0067 ± 0.0006**
	RB	Sheltered	Cíes	6	4	0.733 ± 0.155	0.0012 ± 0.0002
Outer Bay	RB	Sheltered	Ons	6	3	0.600 ± 0.215	0.0007 ± 0.0003
	RB	Sheltered	Roncudo	6	15	0.800 ± 0.172	0.0029 ± 0.0001
	RB	Sheltered	Arealonga	6	0	0.000 ± 0.000	0.0000 ± 0.0000
			**TOTAL**	**24**	**42**	**0.899 ± 0.037**	**0.0078 ± 0.0009**
	SU	Exposed	Cíes	6	4	0.800 ± 0.172	0.0008 ± 0.0002
Outer Bay	SU	Exposed	Ons	6	6	0.933 ± 0.122	0.0016 ± 0.0003
	SU	Exposed	Roncudo	6	2	0.600 ± 0.215	0.0005 ± 0.0002
	SU	Exposed	Arealonga	6	4	0.600 ± 0.215	0.0008 ± 0.0004
			**TOTAL**	**24**	**43**	**0.942 ± 0.029**	**0.0079 ± 0.0010**

[a] number of segregating sites; [b] haplotype diversity; [c] nucleotide diversity per site
